# Supplementary material for: Extra-pair paternity drives plumage colour elaboration in male passerines
Source: PLoS One. 2022 Aug 22;17(8):e0273347. doi: 10.1371/journal.pone.0273347 (PMC9394826; doi:10.1371/journal.pone.0273347)
Supplement: S2 File — (HTML) [file pone.0273347.s002.html]

originaleppcolour


Code 

- Show All Code
- Hide All Code

# originaleppcolour

#### 2022-07-18

# 1 Libraries

```
library(ape)
library(phytools)
```

```
## Loading required package: maps
```

```
library(parallel)
library(geiger)
library(nlme)
library(reshape2)
library(ggplot2)
library(scales)
```

```
## 
## Attaching package: 'scales'
```

```
## The following object is masked from 'package:geiger':
## 
##     rescale
```

# 2 Helper functions for parallelizing

These are collapsed by default but you can expand them.

```
mclapply2=function(presched){
  function(X,FUN,...) parallel::mclapply(X,FUN,mc.preschedule = presched,mc.cores = parallel::detectCores(),...)
}
mclapplyLB = mclapply2(F)
mclapplyPS = mclapply2(T)
mclapplyxx = mclapplyPS
```

These essentially work the way lapply do.

# 3 Read in data

```
name.data=read.csv("EPPColour.csv")
extra.data=read.csv("lat.csv")

merged.data<-merge(name.data,extra.data, by=c("Species","Species"))

name.data=subset(merged.data,!(epp%in% NA))

ericson="ericson-tree-pruner-37e8d136-2f86-455a-a786-85d39d0c9097/output.nex"
hackett="hackett-tree-pruner-dd98f53a-1c49-4461-804b-1156b30d6e7f/output.nex"


treetype=hackett


trees=read.nexus(treetype)


tips=trees[[1]]$tip.label
tree.names=data.frame(Species=tips)

keep=merge(tree.names,name.data,by="Species")


trees=lapply(trees,\(tree)drop.tip(tree,tree$tip.label[-match(keep$Species, tree$tip.label)]))


pruned.tips=trees[[1]]$tip.label#just checking the length of the pruned tip labels with the length of the trait data
traits=data.frame(keep, row.names=1)

data=traits
results = sapply(trees,\(tree)name.check(tree,data))
ifelse(all (results=="OK"),"all OK","err somewhere")
```

```
## [1] "all OK"
```

```
attach(data)
```

This step creates all four model. This step can be SLOW, hence
parallelizing.

```
epp.male.dale<-mclapplyxx(trees,\(tree){
  gls(male.dale~epp + Lat_abs, correlation = corBrownian(phy = tree), data = data, method = "ML")}
)
```

```
epp.female.dale<-mclapplyxx(trees,\(tree){
  gls(female.dale ~ epp + Lat_abs, correlation = corBrownian(phy = tree), data = data, method = "ML")}
)
```

```
epp.male.di.dale<-mclapplyxx(trees,\(tree){
  gls( di.dale ~ epp+Lat_abs, correlation = corBrownian(phy = tree), data = data, method = "ML")}
)
```

```
epp.male.di.dale.abs<-mclapplyxx(trees,\(tree){
  gls(di.dale.abs ~ epp+Lat_abs, correlation = corBrownian(phy = tree), data = data, method = "ML")}
)
```

# 4 Results

You can expand this section to see the helper functions. They’re
necessary to run the code but not to understand results.

```
require(reshape2)
anovasummaries=function(anovas){
  result = (t(sapply(anovas,function(summary)summary[,3]))) |> as.data.frame()
  names(result)=rownames(anovas[[1]])
  melt(result,value.name = "pvalue",variable.name =  "variable",id.vars=NULL)
}
glssummaries=function(glss){
  result = (t(sapply(glss,function(gl) gl$coefficients  )  )) |> as.data.frame()
  names(result)=names(glss[[1]]$coefficients)
  head(result)
  melt(result,value.name = "estimate",variable.name =  "variable",id.vars=NULL)
}
eighthtrans = trans_new(
  name="eighth",
  transform=function(x)x^(1/8),
  inverse=function(y)y^8
)
aggregatesummary=function(ag){
  toprint = t(ag[,-1]) |>as.data.frame()
  names(toprint)=ag[,1]
  toprint
}
ggboxplot.pvalue=function(df.orig,title=NULL){
  if(is.null(title)) title = deparse(substitute(df.orig))
  p=ggplot(df.orig,aes(x=variable,y=pvalue))+
    geom_boxplot()+
    coord_trans(y=eighthtrans)+
    scale_y_continuous(breaks=c(0,.001,.005,.01,.05,.1,.25,.5,1))+ggtitle(title)
  print(p)
  cat(c("Numerical summaries of the posteriors (matches the graphs above) \n\n"))
  (aggregate(pvalue~.,df.orig,FUN=summary)) |> aggregatesummary() |>print()
}
ggboxplot.estimate=function(df.orig,title=NULL){
  if(is.null(title)) title = deparse(substitute(df.orig))
  p=ggplot(df.orig,aes(x=variable,y=estimate))+
    geom_boxplot()+
    ggtitle(title)+facet_wrap(~variable,scales="free")
  print(p)
  cat(c("numerical summaries of the posteriors (matches the graphs above) \n\n"))
  aggregate(estimate~.,df.orig,FUN=summary)|>aggregatesummary() |>print()
}
credibleIntervals=function(df.orig,alpha=.05){
  p=c(alpha/2,1-alpha/2)
  qf = \(x) quantile(x,probs=p)
  result = aggregate(estimate~.,df.orig,FUN=qf)
  sig = ifelse(apply(sign(result[,2]),1,prod)>0,"*","")
  result=data.frame(result,sig)
}

posteriors = function(modelname){
  tit=modelname
  i=which(names(allmodels)==tit)
  
  #create the boxplot and aggregate info
  estimates = allmodels[[i]] |> glssummaries()
  ggboxplot.estimate(estimates,title = tit)
  
  cat("\n\n ### 95% Credible Intervals: \n")
  credibleIntervals(estimates) |> as.data.frame() |> print()
}

allmodels = list(epp.male.dale=epp.male.dale,
                 epp.female.dale=epp.female.dale,
                 epp.male.di.dale=epp.male.di.dale,
                 epp.male.di.dale.abs=epp.male.di.dale.abs)
```

## 4.1 Posterriors of Slopes with credible intervals

These are the posterior distributions from each of the models. After
each plot is credible intervals.

Afterward, if you’re curious, there’s the posterior of pvalues from
the ANOVA. However, we tend to not mix p-values in Bayesian analyses
because they’re they’re philosphically incompatible.

### 4.1.1 epp.male.dale

```
posteriors("epp.male.dale")
```

```
## numerical summaries of the posteriors (matches the graphs above) 
## 
##         (Intercept)        epp     Lat_abs
## Min.       14.22983 -0.5236483 -0.47189966
## 1st Qu.    44.97178  0.1206358 -0.16376028
## Median     47.41593  0.1741945 -0.06237303
## Mean       47.40501  0.1771753 -0.05973850
## 3rd Qu.    50.20759  0.2305131  0.01830682
## Max.       62.53533  0.4897468  1.24232533
## 
## 
##  ### 95% Credible Intervals: 
##      variable estimate.2.5% estimate.97.5% sig
## 1 (Intercept)    37.9475172     55.3018438   *
## 2         epp     0.0357642      0.3415958   *
## 3     Lat_abs    -0.3496369      0.3358676
```

### 4.1.2 epp.female.dale

```
posteriors("epp.female.dale")
```

```
## numerical summaries of the posteriors (matches the graphs above) 
## 
##         (Intercept)          epp      Lat_abs
## Min.       37.60370 -0.752155402 -0.398197272
## 1st Qu.    44.96213 -0.026314373 -0.060681108
## Median     46.63367  0.002189477  0.009281688
## Mean       46.75420  0.001217777  0.008563786
## 3rd Qu.    48.22535  0.033620385  0.080428314
## Max.       57.78055  0.298879528  0.730889431
## 
## 
##  ### 95% Credible Intervals: 
##      variable estimate.2.5% estimate.97.5% sig
## 1 (Intercept)   40.73480835    53.33706726   *
## 2         epp   -0.11029156     0.09448823    
## 3     Lat_abs   -0.25495281     0.26965211
```

### 4.1.3 epp.male.di.dale

```
posteriors("epp.male.di.dale")
```

```
## numerical summaries of the posteriors (matches the graphs above) 
## 
##         (Intercept)        epp     Lat_abs
## Min.    -41.1273005 -0.1117147 -0.80288705
## 1st Qu.  -2.4238083  0.1208213 -0.17038558
## Median    0.6081155  0.1632997 -0.05976077
## Mean      0.6508168  0.1759575 -0.06830229
## 3rd Qu.   3.4006593  0.2270523  0.03055778
## Max.     19.8738896  0.5720377  1.56990328
## 
## 
##  ### 95% Credible Intervals: 
##      variable estimate.2.5% estimate.97.5% sig
## 1 (Intercept)  -11.26775357    12.58457186    
## 2         epp    0.03410586     0.35540174   *
## 3     Lat_abs   -0.59475561     0.43815181
```

### 4.1.4 epp.male.di.dale.abs

```
posteriors("epp.male.di.dale.abs")
```

```
## numerical summaries of the posteriors (matches the graphs above) 
## 
##         (Intercept)        epp     Lat_abs
## Min.    -38.5257063 -0.1118108 -0.76557145
## 1st Qu.   0.1722344  0.1053784 -0.10440561
## Median    3.1933184  0.1423961 -0.03399716
## Mean      2.4115101  0.1606801 -0.01296650
## 3rd Qu.   5.1084838  0.2075452  0.04955921
## Max.     21.8776528  0.5657746  1.57190351
## 
## 
##  ### 95% Credible Intervals: 
##      variable estimate.2.5% estimate.97.5% sig
## 1 (Intercept)    -8.5313877      9.6087421    
## 2         epp     0.0476603      0.3345047   *
## 3     Lat_abs    -0.2968140      0.4422772
```

### 4.1.5 Intervals (summarized in a single table)

```
labels=
  c("Male Colour Score",
    "Female Colour Score",
    "Dichromatism (Male-Female Colour Score)",
    "Dichromatism (Absolute Difference)"
    )

allintervals = lapply(allmodels,function(mod)
  glssummaries(mod) |> credibleIntervals() |> as.data.frame())


intervalstoprint = lapply(allintervals,function(interv)interv[2,])

print(treetype)
```

```
## [1] "hackett-tree-pruner-dd98f53a-1c49-4461-804b-1156b30d6e7f/output.nex"
```

```
do.call(rbind,intervalstoprint)[,-1] |> print()
```

```
##                      estimate.2.5% estimate.97.5% sig
## epp.male.dale           0.03576420     0.34159575   *
## epp.female.dale        -0.11029156     0.09448823    
## epp.male.di.dale        0.03410586     0.35540174   *
## epp.male.di.dale.abs    0.04766030     0.33450472   *
```

## 4.2 ANOVAs

These are the pvalues from each of the models. In a Bayesian type
analysis these are not really appropriate, but I’ve included them for
interest’s sake.

```
for (i in 1:length(allmodels)){
  singlemodel = allmodels[[i]]
  tit = names(allmodels)[i]
  mclapplyxx(singlemodel,anova) |> anovasummaries() |> ggboxplot.pvalue(title = tit)
}
```

```
## Numerical summaries of the posteriors (matches the graphs above) 
## 
##          (Intercept)           epp      Lat_abs
## Min.    1.496719e-13 4.630494e-176 2.109739e-99
## 1st Qu. 2.193978e-09  2.767034e-12 1.624455e-05
## Median  3.776388e-08  1.411305e-06 2.428717e-02
## Mean    1.736639e-04  3.837384e-02 1.884292e-01
## 3rd Qu. 7.190102e-07  8.477753e-04 3.250577e-01
## Max.    2.141709e-02  9.689477e-01 9.954901e-01
```

```
## Numerical summaries of the posteriors (matches the graphs above) 
## 
##          (Intercept)          epp      Lat_abs
## Min.    9.377859e-16 1.767453e-69 2.035586e-93
## 1st Qu. 1.199749e-11 2.829447e-02 5.806484e-04
## Median  2.653753e-10 1.682660e-01 4.410465e-02
## Mean    4.655871e-05 2.825982e-01 1.915986e-01
## 3rd Qu. 8.671537e-09 4.796115e-01 2.899669e-01
## Max.    2.760853e-02 9.984802e-01 9.999185e-01
```

```
## Numerical summaries of the posteriors (matches the graphs above) 
## 
##         (Intercept)           epp       Lat_abs
## Min.      0.6757750 3.874354e-175 9.564079e-119
## 1st Qu.   0.7801425  2.501596e-11  1.609460e-05
## Median    0.8058308  1.209542e-05  2.173288e-02
## Mean      0.8081622  2.745124e-02  1.833022e-01
## 3rd Qu.   0.8359622  1.514496e-03  2.920695e-01
## Max.      0.9275808  9.362250e-01  9.980092e-01
```

```
## Numerical summaries of the posteriors (matches the graphs above) 
## 
##         (Intercept)           epp      Lat_abs
## Min.      0.3554593 7.349409e-180 3.177895e-93
## 1st Qu.   0.4865026  7.775196e-12 3.045173e-04
## Median    0.5262847  1.059172e-05 3.484963e-02
## Mean      0.5533848  2.464433e-02 2.006476e-01
## 3rd Qu.   0.6112209  2.110100e-03 3.263120e-01
## Max.      0.8432595  9.460743e-01 9.959373e-01
```

# 5 Graphs in manuscript

## 5.1 The boxplots of slopes

```
posteriors_slope = function(modelname){
  tit=modelname
  i=which(names(allmodels)==tit)
  
  #create the boxplot and aggregate info
  estimates = allmodels[[i]] |> 
    glssummaries() |> 

    ggboxplot.estimate(estimates,title = tit)+ylab("Estimate")
  
}

slopesonly = lapply(allmodels,function(mod){
  glssummaries(mod)|> subset(variable!="(Intercept)" & variable!="Lat_abs")
}) 

slopesonly2=do.call(rbind,slopesonly)


slopesonly2$variable =  sapply(labels,rep,rep,times=length(trees)) |> as.vector()


ggplot(slopesonly2,aes(x=variable,y=estimate))+
  geom_boxplot()+
  geom_hline(yintercept=0,color="red")+
  xlab("Posterior Estimate")+ylab("Variable")+
  facet_wrap(variable~.,scales="free",ncol=1)+
  theme(strip.text.x = element_blank())+
  coord_flip()
```

```
#  ggboxplot.estimate(slopesonly2)
```

## 5.2 The regression lines

And the graphs

```
makeplot = function(mod,yvar,ylab,xlab="Extra-Pair Paternity (% Offspring)"){

  params=sapply(mod,coefficients) #3x1000 matrix. cols=intercept/slopes from each tree.
  plot(yvar~epp,
       data=data,
       main="",
  xlab=xlab,ylab=ylab
  )
  
  for (i in 1:ncol(params)){
    abline(params[1,i],params[2,i],col=rgb(0,0,.7,.05))
  }
  points(yvar~epp,data=data,pch=16,col=rgb(.25,.25,.25,.25))
  
}

par(mfrow=c(2,2),mar=c(1,1,0,0)+3,mgp=c(2,1,0))

plottingvars = data |>subset(select=c(male.dale,female.dale,di.dale,di.dale.abs))

for (i in 1:ncol(plottingvars)){
  makeplot(allmodels[[i]],plottingvars[,i],labels[i])
}
```
