# Supplementary material for: Extra-pair paternity drives plumage colour elaboration in male passerines
Source: PLoS One. 2022 Aug 22;17(8):e0273347. doi: 10.1371/journal.pone.0273347 (PMC9394826; doi:10.1371/journal.pone.0273347)
Supplement: Attachment — Submitted filename: EPP and colour review response_v2.docx [file pone.0273347.s003.docx]

Dear Dr. Stephens,

Thank you for your time and consideration of our manuscript. We thank the editor and reviewer for their constructive comments. We have addressed all comments below and in the manuscript and we believe they have improved the quality of our manuscript. Thank you again and we very much look forward to hearing your response.

Sincerely,

Matt Reudink and co-authors

**Editor’s comments**

**Editor comments	Authors’ response**

| Overall this is a solid study on a topic of wide interest.  There some points of clarification needed in the methods, and assuming that your input trees were largely invariant for the 186 species of the study no new analyses will be needed.  However, if both the branch lengths and the topology vary, I believe you need to follow standard practice with the Jetz group trees and repeat your analysis on a sample of trees. | We thank the editor for the feedback and have re-run the analyses—please see details below. |
| --- | --- |
| 1. Line 112: please define “male like”.  Does it mean high variance among RBG scores from different parts of the body?    I assume that being more “male like” means more colorful or complex patterns, but I am not sure from the description here how exactly it is quantified. | Thank you for this comment. The term "male-like" is used throughout the Dale et al. paper to describe more colorful species. "Female-like" is used to describe less colorful birds. We agree that it is confusing, so in the revised manuscript we removed the term, briefly explain the calculation, assist the reader in locating a more detailed methods description, and offer an interpretation of the scores: **"From these data, Dale et al. (2015) calculated the mean RGB values from 6 plumage patches to generate a single metric that describes the overall color of a given species’ plumage (for more detailed methods see Dale et al. 2015, Methods: Plumage Scores). Higher color scores for both sexes represent species that are more colorful."** |
| You took a sample of 1000 trees and boiled them down to a single tree using maximum likelihood. This leads to several questions:   1. One birdtree.org, Jetz and colleagues  suggest that all comparative analyses of birds be performed using sample of trees from their posterior distribution, and that is becoming standard practice when using the Jetz et al bird trees or the Upham et al. mammal trees.  Why did you do your analysis on a single tree rather than repeating it on a sample? 2. Since you wanted to use a single tree, why didn’t you use the maximum clade credibility tree from Jetz et al.?  If there is a previous study that you can cite for the method that you used to create a ML tree from your sample of 1000, please mention it.  For the 1000 input trees the method you used should produce a nearly identical tree to the MCC tree, but you tree might differ somewhat from the MCC tree of the entire distribution (but see below about toplogy vs branch lengths). 3. It looks like you used the Hackett backbone trees. How did you decide between the two potential backbone trees at birdtree.org? | We thank the reviewer for pointing this out.  Following the editor’s suggestion to point 1, the analysis was rerun on 1000 trees from Hackett and 1000 trees from Ericson. Posterior distributions of slopes from each of the 1000 GLS models from each backbone were analyzed and the text updated.  The text was updated showing the results from both the Hackett trees and Ericson trees, which both provided results with a similar message.  By following the reviewer’s concern regarding point 1, by analyzing the 1000 trees, we believe that we no longer need to address point 2) as the inadequacies of a single tree have been addressed through the reviewer’s first suggestion.  Regarding point 3, we ran the analysis on both backbones and obtained similar results, both of which are now presented in the manuscript. |

**Reviewer #1 comments	Authors’ response**

| The authors address a fundamental hypothesis in avian sexual selection that has yet to be tested with a large and strong dataset. They make use of two publicly available databases and find convincing support for the idea that EPP rates correlate with male plumage elaboration and sexual dichromatism. The study is concise, direct, and strong. I have no issues with the manuscript framing, methods, or results. Well done. | We thank the reviewer for the positive and encouraging feedback. |
| --- | --- |
